# Hints to Young Practitioners—No. VI

**Published:** 1873-05

**Authors:** 

**Affiliations:** Savannah, Georgia


### HINTS TO YOUNG PRACTITIONERS—No. VI.

BY SENEX.

*De minimis* NON *curat lex* is the lawyer's maxim. *De minimis* CURAT *medicina* should be ours. An eminent accoucheur tells us that he once lost a patient by entering her room without knocking. It was a case of confinement, and he surprised her on the close stool. The shock induced fever from which she did not recover.

Let us consider some of the minor points in the conduct of natural labor: You are engaged to a case. It would be well to see your patient once a week during the last month, to give proper directions about diet and exercise and the general condition. Let the nipples be hardened with an astringent, for which purpose this formula does well: ℞—Tannin, ʒ ss; Alum, ʒ ij; Santa Cruz Rum and water, each, ℥ ij. Applied every night and morning. The time has come and you are summoned.

Some, especially multiparous, put off sending as long as they can. It has been my fortune several times to get there a little too late. It is best to be very punctual. You make digital examination, and if you find no dilatation of the os you may leave for a short time, especially if it be a primiparous case; but don't be long away. There is vast comfort in the presence of the Doctor, though you need not be all the time in the room.

Presently you have the question of chloroform to decide. By all means administer it in the second stage, but not before. I think it retards labor if given before the powerful expulsive throes have begun. Give it immediately on the approach of pain, and take away the handkerchief as soon as the pain is over. The child is born by the natural powers purely, and your office is passive. You may or may not "support the perineum," as it is called. The nurse takes the child, (see that it is wrapped in something warm,) and you proceed to deliver the after-birth. Here your functions are more active. If you wait for nature to do the work, you will often wait many a weary hour.

Churchill's method is the best. Keep one hand over and upon the uterus, which will be hard as a ball under it. Within five minutes, or ten at most, you cöoperate with contraction, (which contraction is manifested by more or less hardening,) and with the fingers you squeeze out the placenta, making reasonable traction at the cord with the other hand, and directing the woman to bear down. If the uterus is well contracted, you can not possibly do harm by traction. The worst that could happen would be tearing away the cord ; but beware of any violence or force, especially with an uncontracted womb. If you have hour-glass contraction you must introduce your hand into the upper compartment whilst the womb is relaxed, and gathering the mass in your hand withdraw it, making firm outside pressure. Here I will say that the dorsal posture, from the second stage out, is most convenient. The French prefer it to the English and American left side.

So of the binder, the French attach no importance to it. Out of deference to custom, it may be best to have it applied. I leave it to the nurse.

You direct a teaspoonful of paregoric for after pains, with discretion to repeat it if required. Baby is put to the breast early, nurse having privilege to give a little sweetened water besides. I believe in the old-fashioned custom of giving a dose of castor oil on the third day. Make nurse and husband keep watch and ward over the chamber for a week, keeping away the curious and the officious.

SAVANNAH, GEORGIA, May, 1873.

